# Microstructure and Properties of Sm_2_O_3_ Micro-Dispersed Tungsten-Based Alloy and Its Sintering Evolution

**DOI:** 10.3390/ma18214973

**Published:** 2025-10-31

**Authors:** Song Ye, Ping Wang, Zhiqiang Cui, Ningfei Zhang, Yuhao Wang, Zhenyi Huang

**Affiliations:** 1 School of Metallurgical Engineering, Anhui University of Technology, Ma’anshan 243002, China; yesong20250409@163.com (S.Y.); wangping@ahut.edu.cn (P.W.); zhangningfei@ahut.edu.cn (N.Z.); wyh_02200012@163.com (Y.W.); 2 Anhui Low-Carbon Metallurgy and Process Control Engineering Research Center, Ma’anshan 243002, China; 3 BYD Company Limited, Shanwei 516600, China; 18885652187@163.com

**Keywords:** tungsten, Sm_2_O_3_, core–shell structure, microstructure and properties, sintering evolution

## Abstract

Tungsten (W) is regarded as the most promising plasma-facing material in thermonuclear fusion reactors due to its excellent properties, such as high strength, a high melting point, and a low sputtering rate. However, its low-temperature brittleness, recrystallization embrittlement, and irradiation embrittlement seriously limit the practical application of W. In this research, the properties of tungsten-based materials were improved by introducing second phases into W. Core–shell composite powders with W particles as core and Sm(OH)_3_ thin films as shell were prepared by electroless plating, and sintered by spark plasma sintering (SPS) to obtain bulk. After sintering, the Sm(OH)_3_ shell transformed into the Sm_2_O_3_ phase with a different size, mainly distributed at W grain boundaries. The average size of W grains in the composite material was smaller than that of pure W sintered bulk due to the pinning of W grain boundaries by Sm_2_O_3_, while the porosity of the composite is reduced. Compared with pure W sintered bulk, the composites exhibited better mechanical properties and radiation resistance; although the thermal conductivity decreased somewhat, it still maintained a high level. With the increase in sintering temperature and pressure, the evolution of core–shell powders during the sintering process could be simplified into six stages, which occurred approximately in sequence.

## 1. Introduction

A tokamak, as a magnetic confinement ring system, provides the possibility of realizing thermonuclear fusion energy. The selection of materials for each component in the Tokamak device is very important. Among them, plasma-facing materials (PFMs) are mainly used in the first wall, tritium breeder, and divertor or components of the Tokamak nuclear fusion reactor, which directly bear the impact of high-energy particles from fusion and must resist the combined action of multi-field coupling, such as high thermal load, high stress, neutron radiation, and plasma [1]. Materials researched extensively in the International Thermonuclear Experimental Reactor (ITER) program and other large experiments include tungsten (W), beryllium (Be), and carbon (C) [2]. W plays an important role in modern national defense, the atomic energy industry, electric light sources, and other application fields because of its excellent high-temperature strength, high melting point (≈3695 K), excellent thermal conductivity (≈160 W·m^−1^·K^−1^), and low hydrogen retention characteristics [3]. In the field of PFMs, W applications have gradually moved from laboratory research to the deployment of key components of nuclear fusion devices. ITER diverters have been identified as using all-tungsten armor materials, and the W Environment in Steady-state Tokamak (WEST) has been successfully operated. With the advance of the ITER project, tungsten-based materials have officially established their dominant position in PFMs. However, defects such as intrinsic brittleness, recrystallization embrittlement, and radiation embrittlement limit the application of W in PFMs [4,5].

The current industrial applications mainly include pure tungsten sintering, fiber reinforced tungsten-based alloy, and dispersion strengthened tungsten-based alloy technology routes, in which dispersion strengthened tungsten-based alloy is achieved through the introduction of oxide/carbide particles in the tungsten matrix, synchronous improvement of material performance [6]. Common dispersed phases include Y_2_O_3_, La_2_O_3_, TiC and ZrC [7]. Rare earth oxides (REO) are considered the preferred strengthening phase for improving W properties due to their high thermal stability and melting point higher than W recrystallization temperature [8,9,10,11]. Compared with pure W, grain refinement and second-phase strengthening of W-based materials dispersed with REO are considered to be two of the main reasons for weakening the embrittlement effect [8,9,10]. Samarium oxide (Sm_2_O_3_), an important rare-earth oxide, exhibits excellent high-temperature and chemical stability. The atomic radius of samarium element is 185 pm, and the absorption cross section of its isotope for thermal neutrons is about 40,800 b, which can effectively capture energetic particles produced in the irradiation process [12]. Yuan, M. et al.’s [13] research showed that Sm addition could refine the grain size and weaken the texture of as-extruded magnesium alloy, and significantly improve the strength and ductility of as-extruded alloy. In the research of E. M. Abou Hussein et al. [14], with the increase in Sm^3+^ molar percentage, thermal neutron and gamma attenuation also increased, thus improving the protective performance of the glass against gamma, neutron, proton, and other particles. Therefore, Sm_2_O_3_-dispersed W-based material was selected for further research on its preparation, microstructure, properties, and sintering evolution.

REO-dispersed W-based bulk is mainly prepared by mechanical alloying and chemical methods to produce precursor powders [15,16], and then sintered to obtain the bulk. The preparation method and control method of mechanical alloying are relatively simple, but a small amount of impurities is inevitably introduced during the ball milling process, and too long a ball milling time will lead to serious agglomeration of composite powders, resulting in poor sintering activity of powders. At the same time, the compressibility of the powders is significantly reduced due to work hardening, which leads to higher sintering densification difficulty [17]. Among chemical methods, the wet chemical method has become the preferred method for preparing uniform nano-powder because of its obvious advantages, such as simple process, high preparation efficiency, and low cost [17,18,19,20]. In general, the basic process for preparing REO-doped W-based powders by wet chemical methods can be divided into two steps [9,10]. First, a soluble salt containing W reacts with a reducing agent in solution to form hydrated tungsten oxide on the surface of REO, or a soluble salt containing W and a soluble salt containing rare earth react with a reducing agent to form hydrated tungsten oxide and a rare earth hydroxide mixture. Then, hydrated tungsten oxide or rare earth hydroxide is reduced to W and REO, respectively, by hydrogen reduction at high temperature. However, due to the complex relationship between hydrogen reduction process and precursor powders composition has not been fully clarified, the use of general hydrogen reduction process may lead to incomplete hydrogen reduction phenomenon, resulting in residual hydrated oxide or hydroxide in the composite powders, which will release H_2_O (g) during the subsequent sintering process, thus increasing the porosity of the sintered bulk material, which is not conducive to the densification of the sintered bulk [10]. By optimizing the wet chemical preparation process, REO-doped W-based powders can be prepared without a hydrogen reduction step, or hydration products can be controlled on the outer surface of composite powders. Based on this, a layer of rare earth hydroxide shell was coated on the surface of W powders by the solid–liquid chemical method (electroless plating) in this research. The core–shell structure could effectively avoid the direct contact of “core” during the sintering process and inhibit the grain growth of “core” during the sintering process, thus bringing good sinterability to the powders.

The feasibility and effectiveness of Sm_2_O_3_-dispersed W-based bulk materials prepared by core–shell W-based composite powders with W particle as core and Sm(OH)_3_ as shell were analyzed. Sm(OH)_3_ was coated on the surface of pure W powders by the electroless plating method, and then, respectively, the pure W powders and core–shell powders were sintered by spark plasma sintering (SPS) to prepare bulk, with the pure W powders sintered bulk serving as a reference sample. The microstructure and properties of pure W and core–shell powders were compared and analyzed, demonstrating the feasibility and effectiveness of the above methods. And the sintering evolution process of core–shell powders was analyzed using a double-sphere sintering model.

## 2. Experimental Process

### 2.1. Preparation of Precursor Powders

Before electroless plating, the W powders (purity > 99.9%, average particle size 0.5~2 μm) were pretreated by coarsening, activation, and sensitization, so as to improve the surface state of the powders and improve the bonding performance between the W powders and their surface shell in the subsequent plating process. The pretreatment process was as follows: firstly, the powders were coarsened with 3%HCl solution for 12 h, then activated and sensitized with 30 g/L SnCl_2_ + 5%HCl solution for 1 h, then the solid–liquid mixture was separated by vacuum filtration device, and the powders were washed by ultrasonic wave in deionized water and anhydrous ethanol for 3~5 times, and finally dried in a vacuum drying oven at 333 K for 24 h to obtain the pretreated W powders.

**Figure 1:**
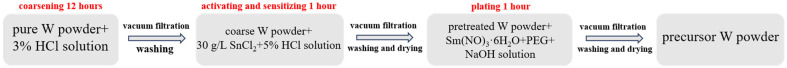
Flow diagram of precursor powder preparation process.

Sm(OH)_3_ was coated on the surface of pretreated W powders using electroless plating. The aqueous solution for preparing Sm(OH)_3_ consisted of the main salt samarium nitrate hexahydrate (Sm(NO)_3_·6H_2_O, Aladdin (Shanghai, China), purity > 99.99%), reducing agent sodium hydroxide (NaOH, Sinopharm (Shanghai, China), purity > 99.5%), stabilizer and dispersant polyethylene glycol (PEG, Sinopharm, purity > 99.98%). In the electroless plating process, the pH value was controlled between 7 and 10 by adjusting the amount of NaOH. Electroless plating was carried out in a constant temperature water bath at 323 K. In order to homogenize W powders and make W powders fully coated, the pretreated W powders were stirred with Sm(NO)_3_·6H_2_O and PEG mixed solution for 0.5 h before adding NaOH solution. During the plating process, NaOH solution was added dropwise to the mixed solution using a constant pressure funnel to promote heterogeneous nucleation of Sm(OH)_3_ precipitate phase, and stirring and shaking were continued until the plating was finished. After plating, the powders were cleaned with deionized water and anhydrous ethanol and finally dried in a vacuum drying oven at 333 K for 24 h. Figure 1 is a flow diagram of the process for preparing precursor powder. The composition of the electroless plating solution for preparing core–shell powders is shown in Table 1.

### 2.2. Powders Sintering

In order to avoid introducing impurities during powders calcination and simplify the preparation process of W-Sm_2_O_3_ sintered bulk, SPS pre-sintering method (SPS-III, Shanghai Chenhua Science Technology Corp., Ltd., Shanghai, China) was used in this research, that is, the calcination of core–shell powders and SPS were combined, and pure W powders and W-Sm(OH)_3_ core–shell powders were sintered by spark plasma sintering in vacuum. The pretreated pure W powders and core–shell powders were placed in graphite molds, respectively, and graphite paper sheets were placed between the powders and the indenter to facilitate removal of the blocks after sintering. The mold was fixed in the SPS equipment for pre-pressing and adjusting the temperature-measuring hole to align with the infrared thermometer, then the furnace door was closed, and the vacuum in the furnace was pumped to below 5 Pa. For common tungsten, solid phase sintering usually needs to reach about 2273 K to have effective densification, but a large number of studies have shown that pressure is a powerful means to reduce sintering temperature and promote densification [21,22]. In addition, the sintering temperature can be reduced by using ultrafine powder or REO-dispersed [23,24,25].

**Figure 2:**
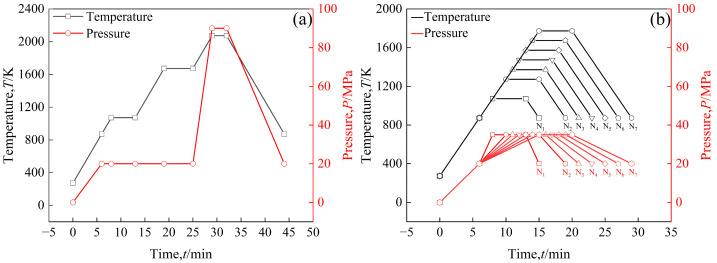
The time–temperature (*t*–*T*) and time–pressure (*t*–*P*) transformation diagram of the sintering process: (**a**) the highest sintering temperature is 2073 K, pure W powders and core–shell powders; (**b**) the highest sintering temperatures of core–shell powders are 1073 K (N_1_), 1273 K (N_2_), 1373 K (N_3_), 1473 K (N_4_), 1573 K (N_5_), 1673 K (N_6_), and 1773 K (N_7_).

In order to increase the density of the sintered bulk, a three-step sintering process, as shown in Figure 2a, was employed, in which the highest sintering temperature was 2073 K to obtain a sintered bulk. The sample was heated to 873 K at a rate of 100 K/min, during which the uniaxial pressure was increased to 20 MPa [26], and then heated to 1073 K at the same rate at 20 MPa and held for 5 min to decompose Sm(OH)_3_ into Sm_2_O_3_. The sample was then heated to 1673 K at a rate of 100 K/min and held for 6 min to release residual gases from the powders. The sample was heated to 2073 K at a heating rate of 100 K/min, during which the uniaxial pressure was increased to 90 MPa, and sintered at this temperature for 3 min. In order to prevent oxidation of the sample, the entire sintering process was carried out under vacuum conditions. During the cooling process, the temperature dropped to 873 K at a rate of 100 K/min, during which the uniaxial pressure dropped to 20 MPa, and then the furnace cooled to room temperature. In addition, in order to reflect the densification process of powders during sintering, time-temperature and time-pressure transition diagrams of sintered powders at different temperatures (1073 K, 1273 K, 1373 K, 1473 K, 1573 K, 1673 K, 1773 K) were also provided, as shown in Figure 2b. The sintering process is shown in Figure 2b, first heating the samples to 873 K at a rate of 100 K/min, during which the uniaxial pressure was increased to 20 MPa, and then heating the samples to a set temperature of 1073~1773 K at a rate of 100 K/min, during which the uniaxial pressure was increased to 35 MPa. The samples were maintained at the set temperature for 5 min., then cooled to 873 K at 100 K/min, during which uniaxial pressure was reduced to 20 MPa, and then cooled to room temperature in the furnace.

### 2.3. Microstructure and Performance Characterization

The microstructure and composition of the powders before and after sintering were investigated by field-emission scanning electron microscope (SEM, TESCAN MIRA3, TESCAN, Brno, Czech Republic) with energy dispersive X-ray spectrometer (EDS, Oxford, UK), X-ray diffractometer (XRD, Rigaku Ultima IV, Rigaku Corporation, Tokyo, Japan) operating with Cu Kα radiation (step size 0.02°, scanning speed 2°/min), SEM with electron backscatter diffraction (EBSD) component (JEOL JSM-7800FPRIME, JEOL, Tokyo, Japan) operating with accelerating voltage of 20 kV (step size 0.2 um), and X-ray photoelectron spectrometer (XPS, Thermo ESCALAB 250XI, Thermo Fisher Scientific, Waltham, MA, USA) operating with Al Kα radiation (operating voltage 12.5 kV, pass energy 20 eV, step size 0.1 eV). An etching solution for etching the polished surface of the sample to reveal the microstructure was prepared using a mixed solution of deionized water (H_2_O, 90 mL), potassium ferricyanide (K_3_[Fe(CN)_6_], 4 g), and potassium hydroxide (KOH, 6 g). Image analysis software (ImageJ, Fiji 2.3.0) was used to estimate the particle size and porosity of the sintered bulk. The density of the sintered bulk at room temperature was measured by a density balance based on Archimedes’ principle.

The ability of the sintered bulk to resist elastic failure was evaluated by the ratio H/E* [27], where H and E* are the hardness and effective Young’s modulus, obtained using a NanoTest Vantage (Micro Materials Ltd., Wrexham, UK). Nanoindentation measurements were carried out on the polished and corroded surfaces of the sintered bulk. The maximum load was 60 mN, and the holding time of the maximum load was 2 s. The nanoindentation effective modulus E* = E/(1 − ν^2^) and hardness H were calculated according to the loading/unloading curves measured by Berkovich indenter with the Oliver–Pharr method [28], where E and ν are Young’s modulus and Poisson’s ratio of the sintered bulk, and Poisson’s ratio of the alloy is 0.28 [29]. An indentation test was carried out on the sintered bulk with an Electric Brinell optical hardness tester (HBRV-187.5), HUATEC Group, Beijing, China. The test force was 980.7 N, and the holding time was 25 s. Crack resistance parameters of the sintered bulk were calculated by F/c^3/2^ [30], where F and c were test forces and crack length. The morphology of the indentation surface was observed by a scanning electron microscope.

λ = α·C_p_·ρ was used to calculate the thermal conductivity λ of sintered bulk at room temperature, where α, C_p,_ and ρ are the thermal diffusion coefficient, specific heat capacity, and density of sintered bulk, respectively.α and C_p_ were measured by laser thermal conductivity analyzer (LFA457) (NETZSCH-Gerätebau GmbH, Selb, Germany) and a synchronous TG-DSC thermal analyzer (STA449F3) (NETZSCH-Gerätebau GmbH, Selb, Germany), respectively. The ability of sintered bulk to resist He^+^ irradiation was evaluated by the plasma surface interaction system (PSIEC) [31]. Polished specimens with a size of 10 mm × 5 mm × 1 mm were irradiated with an irradiation surface of 10 mm × 5 mm. The sample surface was irradiated by a helium ion beam with an ion energy of 30 eV at room temperature. The helium ion dose was 9.918 × 10^24^ ions·m^−2^, the helium ion flux was 5.51 × 10^21^ ions·m^−2^·s^−1^, and the irradiation time was about 30 min.

## 3. Results and Discussion

### 3.1. Powders Characteristics

**Figure 3:**
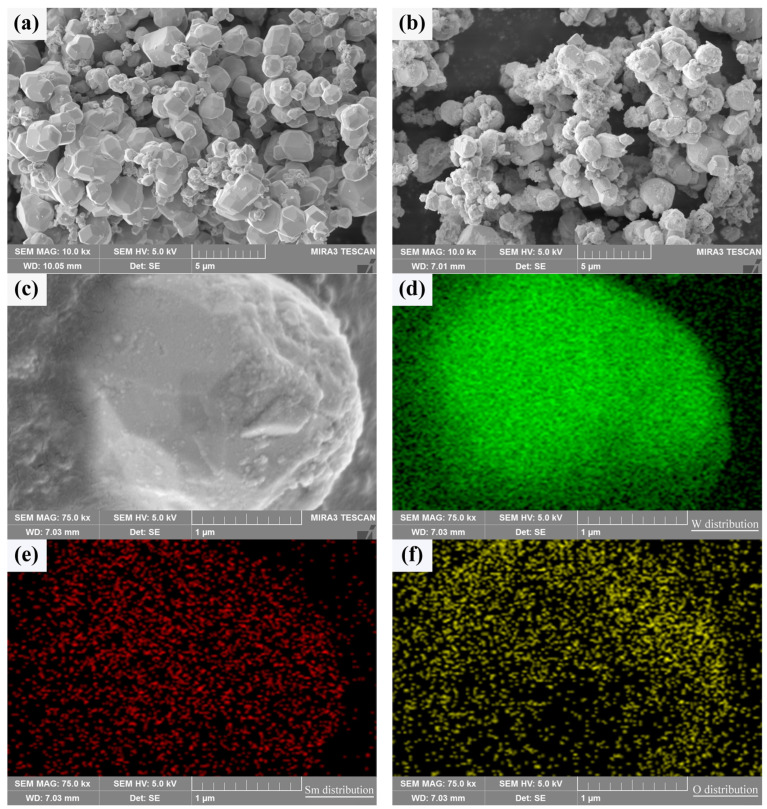
SEM micrograph of the powders and the element distribution of core–shell powder: (**a**) pure W powders; (**b**,**c**) core–shell powders; (**d**–**f**) distribution of W, Sm, and O in core–shell powder.

In order to verify whether Sm(OH)_3_ could be coated on the surface of activated W powders to form a core–shell structure with W particles as the core and Sm(OH)_3_ as the shell, the composition characteristics and element distribution of activated W powders before and after electroless plating were analyzed by SEM and EDS, as shown in Figure 3. It can be seen that both types of powders exhibit some degree of agglomeration. In Figure 3a, there are nearly spherical pure W particles. In Figure 3b, it can be seen that there are a large number of Sm(OH)_3_ particles on the surface of the W-Sm(OH)_3_ core–shell powders, and most areas of the W powders surface are completely covered by Sm(OH)_3_, indicating that Sm(OH)_3_ is successfully coated on the surface of W powders. The enlarged image of a coated particle in Figure 3c and the EDS analysis corresponding to Figure 3d–f show that the particle mainly contains W, Sm, and O, and the distribution of Sm and O elements is almost the same, indicating that Sm(OH)_3_ is relatively uniform and dispersed on the surface of W powders.

In the electroless plating process, excessive NaOH addition caused Sm(OH)_3_ to precipitate from the electroless plating solution containing excessive OH^−^ and coat on the surface of W particles, which could be obtained from Formula (1), so core–shell powders could be prepared, which was consistent with the results shown in Figure 3c–f.

Sm^3+^ + 3OH^−^ → Sm(OH)_3_↓ (1)

However, whether Sm(OH)_3_ successfully coated the surface of W powders or existed independently still needed further confirmation. According to hydrometallurgical principles, solids precipitate from liquids (crystallize), generally in a homogeneous or heterogeneous nucleation manner, depending on the presence of solid particles in the solution and the degree of supersaturation of the solution [32]. When particles are present and the solution is undersaturated, heterogeneous nucleation is dominant. Homogeneous nucleation increased with the increase in supersaturation and gradually dominated [33]. In this research, the supersaturation of the solution was low because NaOH was added dropwise to the solution. Therefore, Sm(OH)_3_ was mainly attached to W particles and crystallized in a heterogeneous nucleation manner, forming a thin film with a certain thickness on the surface of W particles, forming a core–shell structure with W particles as the core and Sm(OH)_3_ as the shell. However, it was undeniable that there might be regions of high supersaturation in the solution, and Sm(OH)_3_ could be crystallized by uniform nucleation.

**Figure 4:**
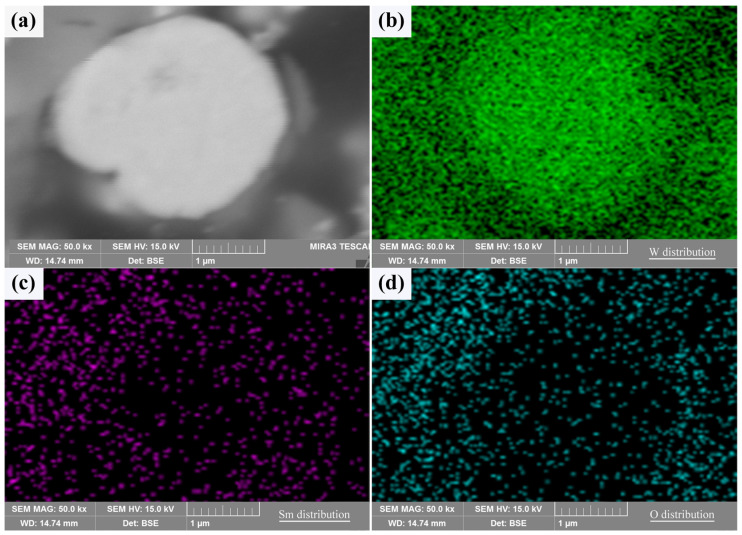
The cross sectional morphology and elemental distribution characteristics of core–shell powder: (**a**) cross section; (**b**–**d**) W, Sm, and O distributions along the cross section.

A typical cross section of the core–shell powder can be used to demonstrate the Sm(OH)_3_ coating state, as shown in Figure 4. The Sm(OH)_3_ coating on W powder was successful due to the overlap of Sm and O-rich regions around the W powder. Since the W powders used in the experiment were not perfectly spherical, the unevenness of the powder’s surface led to the uneven distribution of Sm(OH)_3_ coating on the surface, resulting in the uneven distribution of Sm and O on their surface, as shown in Figure 3 and Figure 4. Therefore, it was difficult to produce a clear cross section by mechanical grinding and polishing, and the distribution of Sm and O elements could also be seen in the cross section.

**Figure 5:**
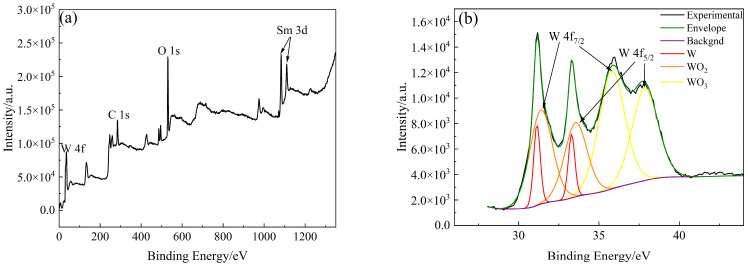

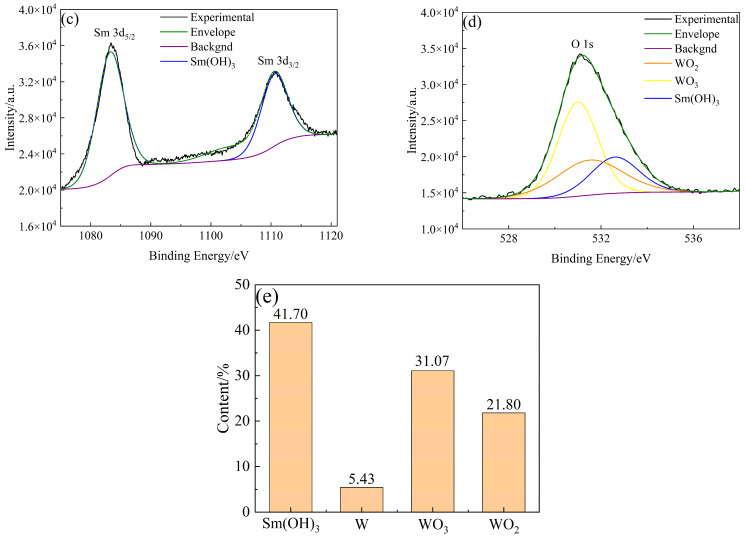
XPS analysis results of W-Sm(OH)_3_ core–shell powders: (**a**) XPS survey spectra; (**b**) W 4f; (**c**) Sm 3d; (**d**) O 1s; (**e**) photoelectron spectroscopy fine spectrum analysis results of W-Sm(OH)_3_ core–shell powders.

The types and contents of elements and compounds on the surface of W-Sm(OH)_3_ core–shell powders were analyzed by XPS. The analysis results are shown in Figure 5. The electron binding energy of the W element (4f_7/2_ characteristic peak) mainly referred to the research results of Kyunghoon Jeong et al. [34] and Luis Díaz-Ballote et al. [35]. The electron binding energies of W 4f_7/2_, WO_2_ 4f_7/2_, and WO_3_ 4f_7/2_ were 31.12 eV, 31.6 eV, and 35.8 eV, respectively. The electron binding energy of the Sm element (3d_5/2_ characteristic peak) mainly referred to the research results of Yin L. X. et al. [36], the electron binding energy of Sm(OH)_3_ 3d_5/2_ was 1183.2 eV. The electron binding energy of the O element (1s characteristic peak) mainly refers to the research results of Pankaj Kumar et al. [37], Rúbia Young Sun Zampiva et al. [38] and K. Veena et al. [39], the electron binding energy of WO_3_ 1s, WO_2_ 1s and Sm(OH)_3_ 1s were 531.0 eV, 531.5 eV and 532.6 eV. The surface of the core–shell powders mainly contained three elements: W, Sm, and O. The main compounds and elements present were Sm(OH)_3_, W, WO_3_, and WO_2_, with their respective contents being 41.70 at%, 5.43 at%, 31.08 at%, and 21.80 at%. XPS analysis results showed that Sm(OH)_3_ was successfully coated on the surface of W powders by electroless plating.

**Figure 6:**
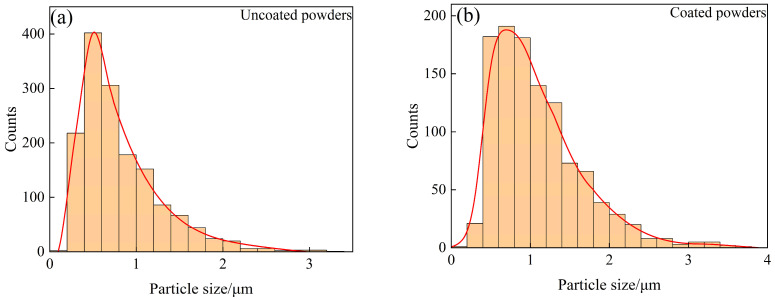
Particle size distribution characteristics of uncoated and coated powders: (**a**) uncoated powders; (**b**) coated powders.

In order to further verify whether Sm(OH)_3_ was successfully coated on the surface of W powder, SEM photos of activated W powders and core–shell powders were selected from 10 random fields, respectively (more than 1000 particles were counted for each powder). The size of these particles was measured by the manual linear intercept method in ImageJ, and the distribution characteristics were analyzed. The results were shown in Figure 6. The average particle size of activated W powders and core–shell powders was 0.848 ± 0.514 μm and 1.100 ± 0.565 μm, respectively. The particle size distribution characteristics of activated W powders and core–shell powders are similar. In contrast, the particle size distribution curve of core–shell powders shifts to the direction of increasing particle size, indicating that Sm(OH)_3_ was successfully coated on the surface of W powders. Combined with Figure 3 and particle size analysis, it could be inferred that both pure W powders and core–shell powders were irregularly spherical, which might lead to certain interparticle forces and exhibit relatively poor fluidity. However, it was not difficult to find that the Sm(OH)_3_ particles coated on the surface of W powders were small enough to fill the pores between W powders, which might reduce friction and adhesion between W powders. This was helpful to improve the apparent density and fluidity of the powders [40,41].

### 3.2. Microstructure and Properties of Sintered Bulk

#### 3.2.1. Microstructural Characteristics

**Figure 7:**
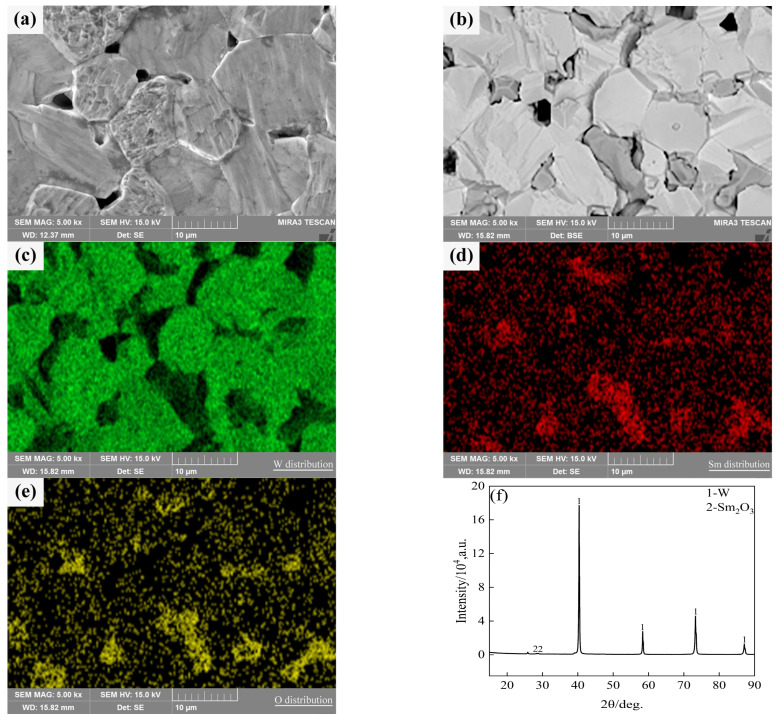
SEM micrograph of the sintered bulks and the element distribution and phase compositions of the sintered bulk from core–shell powders: (**a**) sintered bulk fabricated from pure W powders; (**b**) sintered bulk fabricated from core–shell powders; (**c**–**e**) W, Sm and O distributions in the sintered bulk from core–shell powders; (**f**) phase compositions in the sintered bulk from core–shell powders.

**Figure 8:**
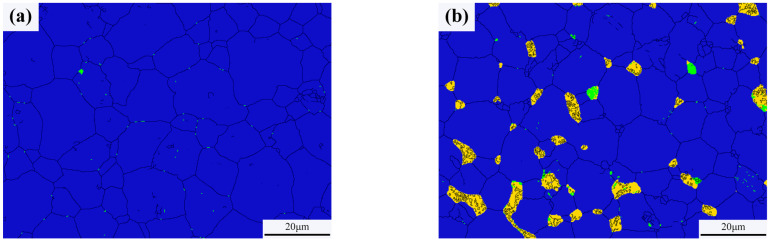
Phase characteristics of the sintered bulks (the black was grain boundaries, the blue was W phase, the yellow was Sm_2_O_3_ and the green was WO*_x_*): (**a**) sintered bulk from pure W powders; (**b**) sintered bulk from core–shell powders.

Typical microstructures of pure W and core–shell powders sintered bulks are shown in Figure 7a,b, respectively. The average W grain size of pure W powders sintered bulk was about 11.69 ± 4.70 μm, and the average W grain size of core–shell powders sintered bulk was about 9.64 ± 3.36 μm, which was significantly lower than the average grain size of pure W. This is due to the pinning effect of Sm_2_O_3_ distributed on W grain boundaries as shown in Figure 7b. From EDS and XRD diagrams of Figure 7c–f, it can be seen that the phase distributed on W grain boundaries is Sm_2_O_3_ instead of Sm(OH)_3_, indicating that the coating powders have undergone a phase transition from Sm(OH)_3_ to Sm_2_O_3_ during sintering. From the microstructure of the sintered bulk core–shell powders shown in Figure 7b–e, it can be seen that Sm_2_O_3_ formed by phase transformation is not uniformly distributed at W grain boundaries or within W grains. Due to the small size of Sm_2_O_3_ inside the W grains, it is difficult to identify them in Figure 7b. Detailed information on the Sm_2_O_3_ phase in sintered bulk core–shell powders can be obtained from EBSD results, as shown in Figure 8b. Compared with the EBSD results of the sintered bulk prepared from pure W powders shown in Figure 8a, it can be seen in Figure 8b that Sm_2_O_3_ is mainly concentrated at W grain boundaries, with a phase size of 4.89 ± 2.54 μm, and is rarely distributed within the W grains. The results are consistent with the distribution characteristics shown in Figure 7b–e. The uneven distribution of Sm_2_O_3_ in the core–shell powders sintered bulk was mainly attributed to the shape and size characteristics of the W particles from which the precursor powders were prepared (Figure 3), resulting in uneven film shapes on the W particles. Using threshold segmentation method in ImageJ, the porosity of two sintered bulks was calculated by SEM photos in different fields. Due to the distribution characteristics of Sm_2_O_3_ in the sintered bulk, the porosity of the sintered bulk was about 4.39%, which was lower than the 7.48% of pure W powders sintered bulk. It could be inferred that the decrease in porosity of the core–shell powders sintered bulk indicates that Sm_2_O_3_ may contribute to pore filling and thus make the sintered bulk more dense.

As can be seen from Figure 8, the relative contents of W and WO*_x_* in the pure W powders sintered bulk are 99.96% and 0.04%, respectively, and the relative contents of W, WO*_x_*, and Sm_2_O_3_ in the core–shell powders sintered bulk are 93.10%, 0.70% and 6.20%, respectively. Because the W element on the surface of W powders was oxidized by alkaline solution during the plating process, the WO*_x_* phase was introduced into the core–shell powders and remained during the sintering process, so that the content of WO*_x_* phase in the core–shell powders sintered bulk was higher than that of pure W.

#### 3.2.2. Thermal Conductivity

Table 2 shows the measured density (ρ), porosity (φ), thermal diffusion coefficient (α), and specific heat capacity (C_p_) at room temperature of pure W and core–shell powders sintered bulk. According to the λ = α·C_p_·ρ formula, the thermal conductivity λ of sintered bulks was calculated and illustrated in Table 2. The α value of the core–shell powders sintered bulk is lower than that of pure W powders sintered bulk, which is due to the different microstructure of the two sintered bulks. As can be seen from Figure 7a,b, the pores that hinder thermal diffusion mainly exist at the W grain boundaries of the sintered bulk [42,43]. However, although the porosity of pure W powders sintered bulk is larger than that of core–shell powders sintered bulk, the thermal diffusion coefficient α of pure W powders sintered bulk is larger than that of core–shell powders sintered bulk. This may be due to the introduced Sm_2_O_3,_ which not only exists as a phase with high thermal resistance, but also generates a significant thermal resistance at grain boundaries due to carrier scattering at the heterogeneous interface between Sm_2_O_3_ and the W matrix. Sm_2_O_3_ also plays a role in refining W grains [44,45]. The smaller the grains are, the more grain boundaries there are, and the more serious the scattering effect is. These effects are more dominant than porosity reduction. The C_p_ value of core–shell powders sintered bulk is lower than that of pure W powders sintered bulk, which is due to the porosity is beneficial to increase the specific heat capacity [46], even if the C_p_ of Sm_2_O_3_ contained in core–shell powders sintered bulk is larger than that of W powders sintered bulk. The measured density ρ of the core–shell powders sintered bulk is lower than that of pure W powders. That is because Sm_2_O_3_ is formed in core–shell powders sintered bulk, and its density is about 8.35 g·cm^−3^, which is much lower than that of W (19.30 g·cm^−3^) [47]. This results in the theoretical density of composites being lower than that of pure W sintered bulk, so the measured density of composites may be lower than that of pure W sintered bulk, although the porosity of composites is lower. It can be seen from the thermal conductivity λ that Sm_2_O_3_ dispersing in W alloy has an adverse effect on its thermal conductivity.

#### 3.2.3. Mechanical Properties

**Figure 9:**
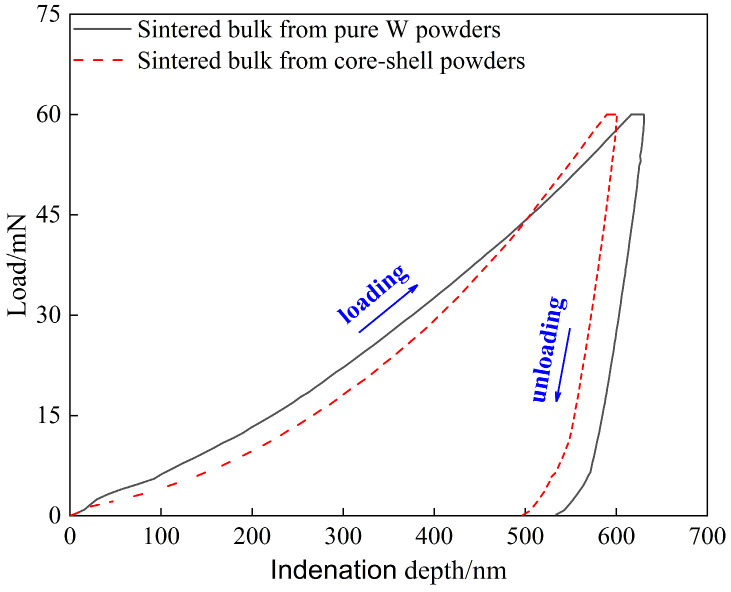
Load–depth curves obtained by nano-indentation measurement conducted on the sintered bulks.

Figure 9 shows the load–displacement (indentation depth) curve of sintered bulk, the area enclosed by the loading and unloading curves indicates plastic deformation energy (W_p_), and the area under the unloading curve indicates elastic deformation energy (W_e_) [48]. The nanoindentation mechanical properties obtained are shown in Table 3, where W_e_% is the elastic recovery rate (the ratio of the elastic deformation energy absorbed relative to the total deformation energy). Some studies showed that brittle materials have elastic failure before plastic deformation, and that the higher the H/E* ratio, the stronger the elastic failure resistance of brittle materials.

Therefore, it can be predicted from Table 3 that at the same maximum load of 60 mN, the elastic failure resistance of the core–shell powders sintered bulk may be stronger than that of the pure W powders sintered bulk, which can also be verified by the W_e_ % values shown in Table 3. However, due to the complexity of evaluating the elastic failure resistance of brittle materials, which includes the effects of indentation depth, applied load, inevitable microstructure inhomogeneity, and experimental errors. Therefore, the Vickers indentation test with 980.7 N load provided further information on the difference in elastic failure resistance between the two sintered bulks.

**Figure 10:**
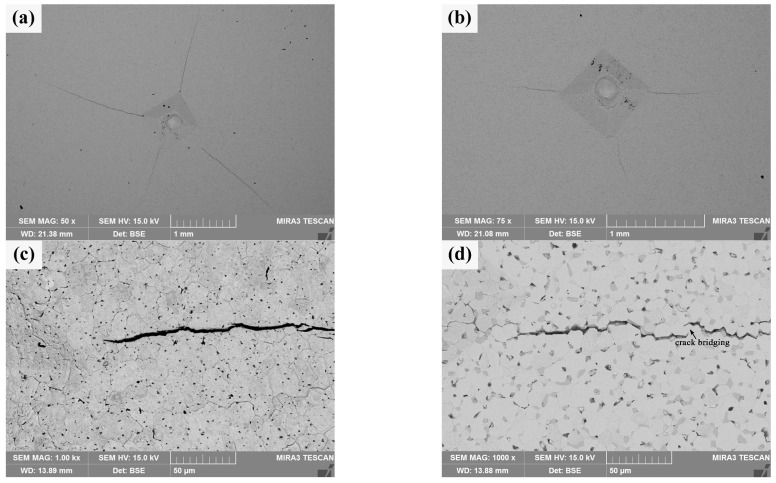
Macro-indentation morphology and micro-crack characteristics of the sintered bulks obtained by the Vickers hardness tester under 980.7 N: (**a**,**c**) sintered bulk from pure W powders; (**b**,**d**) sintered bulk from core–shell powders.

Macro-indentation morphology and micro-crack characteristics of the sintered bulks are shown in Figure 10. It can be seen from Figure 10a that there are obvious cracks at the diagonal corners and near the indentation of pure W powders sintered bulk, but the length of these cracks is uneven, and its average length is about 1.245 ± 0.369 mm; in contrast, the diagonal corners of the indentation on the core–shell powders sintered bulk do not show obvious cracks uniformly, and the crack length is more uniform, as shown in Figure 10b, and its average length is about 0.477 ± 0.082 mm. The crack resistance parameter of the core–shell powders sintered bulk, calculated as F/c^3/2^ is 9.38 × 10^7^ N·m^−3/2^, which is obviously larger than that of pure W (2.90 × 10^7^ N·m^−3/2^). It shows that the former has better resistance to crack initiation and propagation. The crack propagation characteristics at the indentation diagonal of pure W sintered bulk are shown in Figure 10c. The crack propagation path is relatively straight and mainly propagates within W grains. Indentation crack propagation in core–shell sintered bulk is mainly along the W grain boundary, and the propagation path presents an obvious wave shape, which indicates that the deflection effect exists in the propagation process. At the same time, as shown in Figure 10d, there is an obvious bridging phenomenon in the process of crack propagation, which, along with the deflection effect, is beneficial to the improvement of alloy toughness. The main reasons for the higher elastic failure resistance of the sintered core–shell powders than that of pure W powders include finer W grains, lower porosity, and Sm_2_O_3_ toughening.

#### 3.2.4. Irradiation Performance

**Figure 11:**
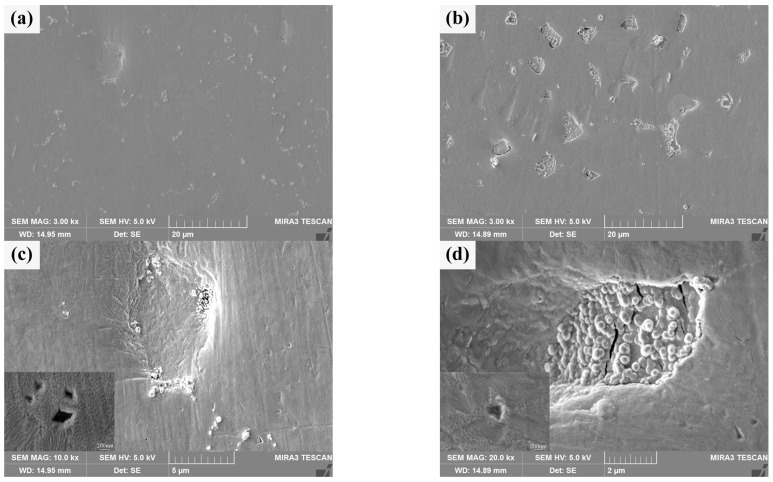
Morphology characteristics of the sintered bulks after He^+^ beam irradiation: (**a**,**c**) sintered bulk from pure W powders; (**b**,**d**) sintered bulk from core–shell powders.

The surface morphology of He^+^ irradiated sintered bulk is shown in Figure 11a,b. There are many white heterogenous products and a few pits on the irradiated damaged surface of pure W powders sintered bulk, and there are few white heterogenous products on the irradiated damaged surface of core–shell powders sintered bulk, but there are many pits and a large number of white heterogenous products in the pits. From the distribution of white heterogenous products after irradiation, it can be seen that there are many undamaged or weakly damaged areas on the W matrix surface of the latter. Figure 11c,d specifically shows different microscopic characteristics for the two sintered bulks. There are obvious granular bulges on the He^+^ irradiated damaged surface and at the boundary of the pits on the pure W sintered bulk, and there is a steep slope from the surface to the bottom of the pits. It is speculated that the formation of pits may be due to the existence of a small amount of tungsten oxide at the grain boundaries of W grains. The sputtering of tungsten oxide during He^+^ irradiation creates initial holes at grain boundaries, and adjacent grains collapse inward due to loss of connection, forming pits. The bonding strength between Sm_2_O_3_ and W matrix in core–shell powders sintered bulk is usually lower than that of pure W grain boundary, and the pits on irradiation-damaged surface collapse in a “cliff” manner. Granular bulges are almost absent in Sm_2_O_3_. At the same time, the high-magnification images of local areas in Figure 11c,d show that the surface tissues of both show wavy structures, which may be caused by near-surface modification [49]. The undulations are more intense in the high-magnification image of Figure 11c, and the defects in the undulations are more pronounced. This implies that the irradiation-induced damage to the surface tissues or the influence is more serious in the pure W powders sintered bulk. Combined with the cracking characteristics of Sm_2_O_3_, it is suggested that the Sm_2_O_3_ or W/Sm_2_O_3_ boundary may be a favorable site for He^+^ accumulation, where He^+^ is “trapped” when it migrates to these sites. As irradiation proceeds, a large amount of He^+^ accumulates and expands at these favorable sites, and damages Sm_2_O_3_ after cracking to a certain extent. It is helpful to reduce the He^+^ concentration in the W matrix and improve the overall radiation resistance [50].

**Figure 12:**
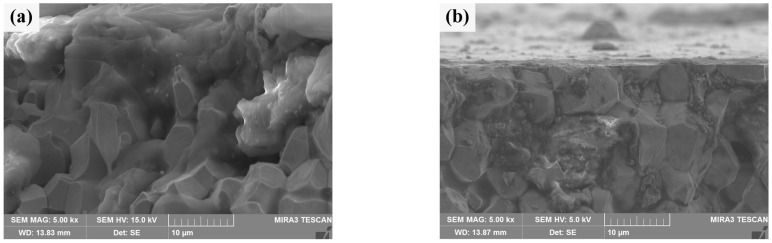
Cross section morphology of the sintered bulks after He^+^ beam irradiation: (**a**) sintered bulk from pure W powders; (**b**) sintered bulk from core–shell powders.

The cross section morphologies of He^+^ irradiation damage of two sintered bulks are shown in Figure 12. By contrast, Figure 12b clearly shows the outline of a section, which is blurred in Figure 12a. In half of the W grains in Figure 12a, there are no obvious grain boundaries between them, and some of them are aggregated together in a “cloud” shape, and holes appear. These are signs of damage to the crystal structure [51]. Figure 12b shows a clearly damaged region, most likely the cracked Sm_2_O_3_ phase depicted in Figure 11. Most of the other areas are grain boundaries that are clearly defined and closely connected between grains. The damage thickness of the W matrix for two kinds of sintered bulks can be obtained by synthesizing the above characteristics. The damage thickness of pure W powders sintered bulk is about 15 μm, while the damage thickness of the W grain of core–shell powders sintered bulk is less than 3 μm. However, Sm_2_O_3_ at W grain boundaries is extremely damaged. It should be noted that, at 30 eV, the projected range of He^+^ in W is nanometres, not micrometers [52]. Therefore, the observed damaged layer was not caused by the direct implantation of He^+^. It was speculated that this was more likely due to irradiation-induced near-surface modification (bubble formation, stress concentration), which made this region more susceptible to mechanical damage or spalling during subsequent cross section sample preparation (cutting, polishing) [53]. The significant difference in damage thickness between the two sintered bulk indicated that Sm_2_O_3_ doping could significantly improve the ability of W alloy to resist radiation-induced damage, that is, the near-surface modification of W-Sm_2_O_3_ sintered bulk was weaker after irradiation, so it was less likely to be damaged during the preparation process [54].

### 3.3. Sintering Evolution Process of Core–Shell Powders

As can be seen from Figure 3, Figure 4, Figure 5 and Figure 6, Sm(OH)_3_ was successfully coated on the surface of W particles in the form of thin films, and Sm(OH)_3_ was transformed into Sm_2_O_3_ during sintering. The Sm_2_O_3_ phase after transformation was mainly distributed at the W grain boundaries in the sintered bulk, contributing to pore filling, thus reducing the porosity and improving the mechanical and radiation properties of the sintered bulk compared with pure W powders. Therefore, it was effective to prepare Sm_2_O_3_ micro-dispersed W-based bulk from core–shell precursor W-based composite powders with W particle as core and Sm(OH)_3_ as shell by SPS without hydrogen reduction treatment. If we want to obtain finer W grains and more uniform Sm_2_O_3_ distribution at W grain boundaries, we can choose nanometer W powders, improve the size uniformity and sphericity of powders, and optimize the electroless plating and sintering process.

**Figure 13:**
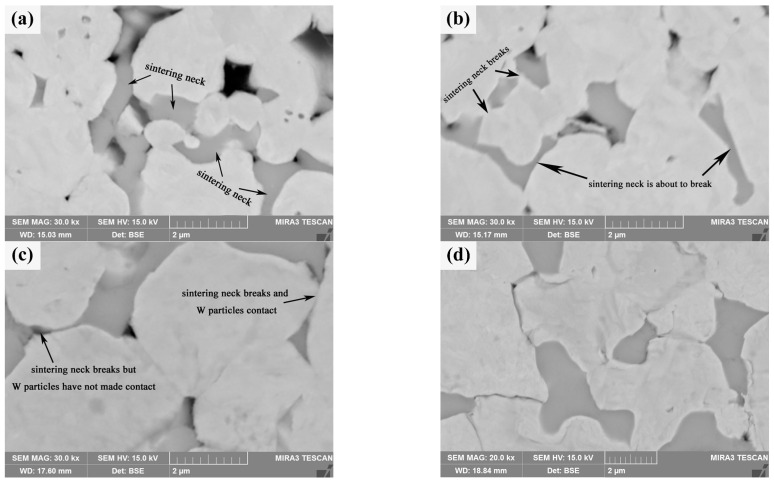
SEM morphology of W-Sm_2_O_3_ sintered bulks at different temperatures: (**a**) 1473 K; (**b**) 1573 K; (**c**) 1673 K; (**d**) 1773 K.

Compared with pure W powders sintering, the evolution of the shell during the core–shell powders sintering process should be paid attention to. After the initial phase of sintering (Sm(OH)_3_ transformed into Sm_2_O_3_), the microstructure evolution of core–shell powders in the intermediate phase is mainly related to the evolution of the Sm_2_O_3_ phase. Figure 13a shows the microstructure of the sintered bulk of core–shell powders at 1473 K, which is mainly divided into Sm_2_O_3_-rich region and Sm_2_O_3_-poor region. Sintering behavior is obvious in both zones, i.e., a sintering neck is formed between powders, and there are a lot of pores on both sides of the sintering neck. It is obvious that the Sm_2_O_3_ shells on the surface of Sm_2_O_3_-rich powders contact each other and form sinter necks under the action of surface energy and sintering pressure, and Sm_2_O_3_ tends to diffuse toward the corners of the sintered necks. Figure 13b shows the microstructure of the sintered bulk of core–shell powders at 1573 K. At this time, only a small amount of Sm_2_O_3_ is found to aggregate in the sintered neck, almost all Sm_2_O_3_ is filled into the pores on both sides of the sintered neck, and the Sm_2_O_3_ sintered neck is in the state of disconnection or about to be disconnected. Figure 13c shows the microstructure of core–shell powders sintered bulk at 1673 K. Due to higher sintering temperature and sintering pressure, the Sm_2_O_3_ sintered neck is broken, and Sm_2_O_3_ fills the pores on both sides of the sintered neck so that the pores on both sides of the sintered neck almost disappear, but W particles in the Sm_2_O_3_-rich region have not contacted and form a W-W sintered neck. Obviously, the dispersion of Sm_2_O_3_ can effectively delay contact between W particles, delay the surface interface between W particles, and form individual particles, thus inhibiting the growth of W grains. Figure 13d shows the microstructure morphology of core–shell powders sintered into a bulk at 1773 K. Sm_2_O_3_ is dispersed at W grain boundaries. The morphology and distribution characteristics are similar to those of Sm_2_O_3_ in core–shell powders sintered bulk at 2073 K in Figure 7b, but the W grain size in sintered bulk at 1773 K is significantly smaller than that at 2073 K, which is due to rapid diffusion of W element and rapid grain growth during 1773~2073 K.

**Figure 14:**
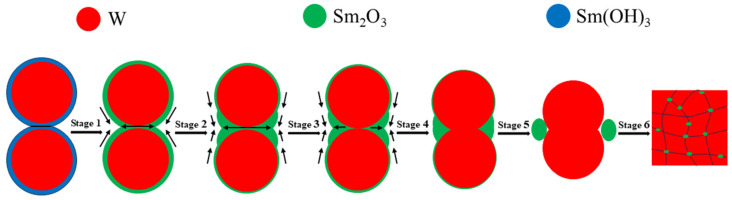
Evolution diagram of core–shell powders during sintering of W-Sm_2_O_3_ alloy.

Wang C. et al. [55] evaluated the densification mechanism of W-La_2_O_3_ sintered bulk by using the two-ball sintering model, focusing on the filling effect of La_2_O_3_ on pores. From the point of view of simplification, the evolution of core–shell powders during sintering process can be divided into six stages, with the increase in sintering temperature and pressure: La(OH)_3_ sintering neck formation, La(OH)_3_ sintering neck transformation into La_2_O_3_ sintering neck, La_2_O_3_ sintering neck growth and deformation, La_2_O_3_ thin film rupture and two W particles started to contact, W sintering neck formation, W sintering neck growth, deformation and La_2_O_3_ spheroidization, these stages were not completely independent, but occurred in an approximate order. Yang H.T. et al. [56] described the macroscopic migration of W-CeO_2_ shell materials from the perspective of microstructure evolution and simulation by using the four-ball sintering model. From a simplified point of view, the formation and growth of sintering necks in the four-ball model were divided into seven stages: moisture release and formation of sintering neck between CeO_2_, growth of sintering neck between CeO_2_ and formation of sintering neck between adjacent CeO_2_ shells, fracture of sintering neck of CeO_2_ shell and reduction in porosity of sintering bulk, pore spheroidization, disappearance of pores, contact between W particles and formation of sintering neck, growth of sintering neck of W and spheroidization of CeO_2_. The variation in sintering neck radius indicated that the core–shell structure of W-CeO_2_ exhibited viscous flow behavior during the sintering process. To sum up, the evolution behavior of core–shell powders during the sintering process is divided into six stages in this research, as shown in Figure 14 [55]. In the first stage (pressure rise period), rare earth hydroxides on the powder’s surface decompose and aggregate due to the initial rearrangement of core–shell powders (sintering pressure plays a major role). With the increase in sintering temperature, the powders contact with each other and begin mass transfer, without obvious densification behavior. In the second stage (1373~1473 K), the formation and growth of the W sintering neck or Sm_2_O_3_ sintering neck occur. Under the influence of surface energy and sintering pressure, the Sm_2_O_3_-rich region tends to diffuse towards the corners of the sintering neck. In the third stage (1473~1573 K), the Sm_2_O_3_ sintering neck shrinks to both corners, and W particles covered with Sm_2_O_3_ gradually approach under the action of sintering pressure. In the fourth stage (1573~1673 K), Sm_2_O_3_ sintering neck breaks, and the W particles covered by Sm_2_O_3_ gradually contact each other under the action of sintering pressure and form a sintering neck. The fifth stage (1673~1773 K) is the growth of the W sintering neck and the spheroidization of Sm_2_O_3_. The spheroidized particles fill the pores on both sides of the sintered neck. The sixth stage (1773~2073 K) is the last stage of sintering, in which W particles grow rapidly, Sm_2_O_3_ near pores fill up pores until they are exhausted, and the remaining pores close gradually. It should be pointed out that the above six stages are not completely independent, and their sequence is not fixed. Two or more stages may occur simultaneously or alternately.

## 4. Conclusions

1. Sm(OH)_3_ was successfully coated on W powders by electroless plating, and core–shell composite powders with W particle as core and Sm(OH)_3_ as shell were obtained. Sm_2_O_3_ phase (4.89 ± 2.54 μm) with different sizes was obtained by sintering Sm(OH)_3_ shell, mainly distributed at W grain boundaries. The average size of W grains in the composites (9.64 ± 3.36 μm) was smaller than that of pure W sintered bulk (11.69 ± 4.70 μm) due to the pinning of W grain boundaries by Sm_2_O_3_.
2. The mechanical properties and radiation properties of core–shell powders sintered bulk were better than those of pure W powders sintered bulk, and the thermal conductivity of core–shell powders sintered bulk was lower than that of pure W powders sintered bulk, but it still maintained a high level.
3. From the point of view of simplification, the core–shell powders sintering process could be roughly divided into six stages, which were not completely independent, but occurred in an approximate order. With the increase in sintering temperature and pressure, Sm(OH)_3_ was transformed into Sm_2_O_3_, the formation and growth of Sm_2_O_3_ sintering neck, Sm_2_O_3_ sintering neck shrank to both corners, the fracture of Sm_2_O_3_ sintering neck and the contact of two W particles, the formation and growth of W sintering neck, as well as the spheroidization of Sm_2_O_3_, W particles growth and pore closure.
4. The porosity of the core–shell powders sintered bulk was about 4.39%, which was lower than the 7.48% of pure W powders sintered bulk. The densification of core–shell powders sintered bulk during the sintering process may be due to Sm_2_O_3_ contributing to pore filling, which could be evaluated by the two-ball sintering neck model.
5. Without hydrogen reduction treatment, Sm_2_O_3_ micro-dispersed W-based bulk prepared from core–shell precursor W-based composite powders with W particles as core and Sm(OH)_3_ as shell was effective.

## Figures and Tables

**Table 1:** Composition of the solution to fabricate W-Sm(OH)_3_ core–shell powders by electroless plating.

Chemical	Formula	Effect	Concentration
Samarium nitrate hexahydrate	Sm(NO)_3_·6H_2_O	Main salt	1.5528 g/L
Sodium hydroxide	NaOH	Reducing agent and pH regulator	>20 mL/L
Polyethylene glycol	C_8_H_14_O_4_	Stabilizing and dispersant agents	1 g/L

**Table 2:** The measured density (ρ, g·cm^−3^), porosity (φ, %), thermal diffusivity (α, 10^−6^ m^2^·s^−1^), specific heat capacity (C_p_, J·g^−1^·K^−1^), and the calculated thermal conductivity (λ, W·m^−1^·K^−1^) of the sintered bulks from pure W powders and core–shell powders.

Sintered Bulks	*ρ*	*φ*	*α*	*C*_p_	*λ*
From pure W powders	17.776 ± 0.14	7.48	56.682 ± 0.902	0.142	143.076 ± 2.540
From core–shell powders	17.556 ± 0.12	4.39	54.219 ± 0.533	0.138	131.358 ± 1.573

**Table 3:** Mechanical properties of the sintered bulks at room temperature evaluated from nano-indentation tests.

Sintered Bulks	*H*/GPa	E*/GPa	H/E*	*W_p_*/nJ	*W_e_*/nJ	*W_e_*%
from pure W powders	7.022 ± 0.123	419.271 ± 1.411	0.017	16.391 ± 1.116	1.891 ± 0.075	10.34
from core–shell powders	7.800 ± 0.045	442.823 ± 1.194	0.018	15.491 ± 0.182	1.934 ± 0.078	11.10

## Data Availability

The original contributions presented in this research are included in the article. Further inquiries can be directed to the corresponding author.
